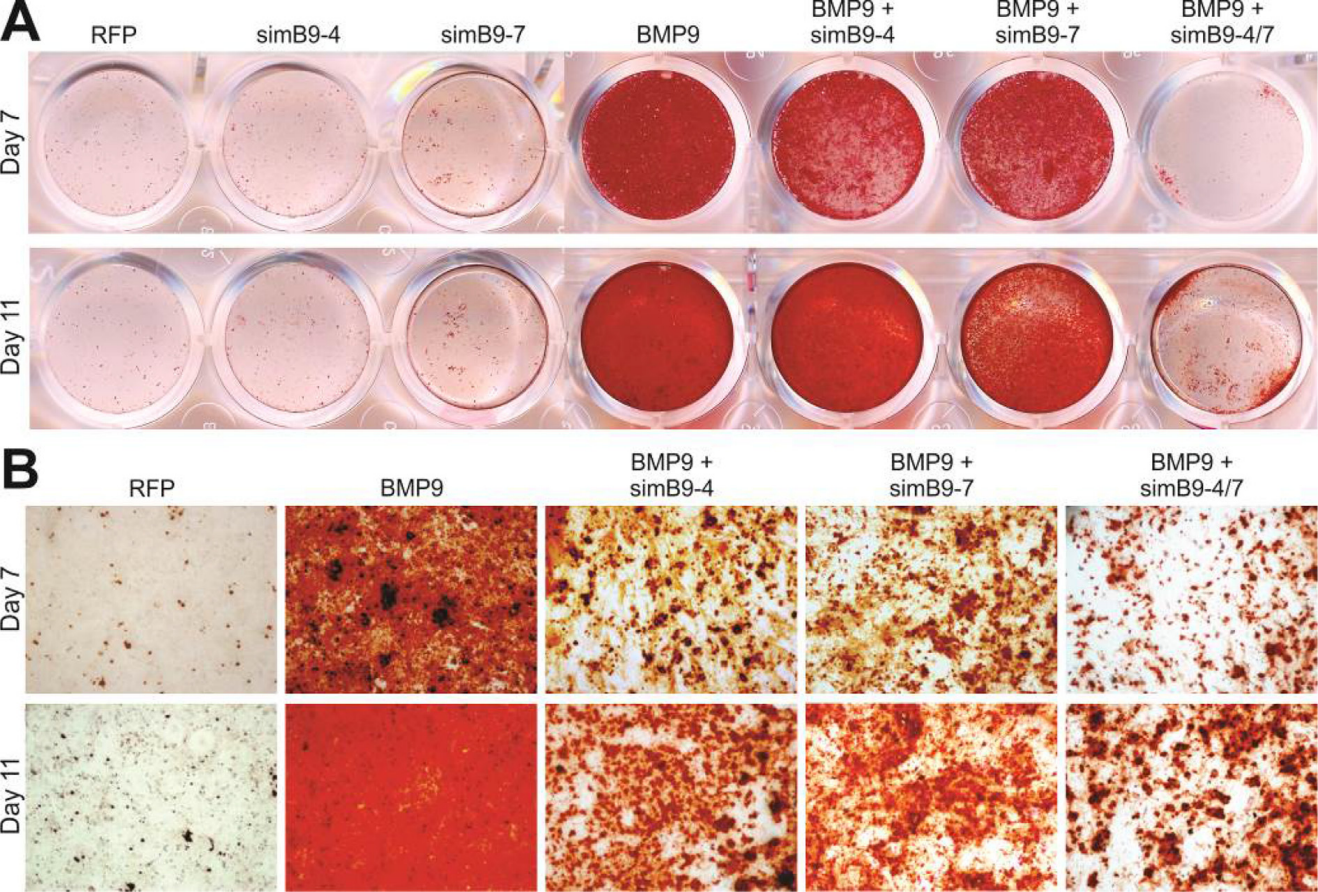# Corrigendum to “Characterization of the essential role of bone morphogenetic protein 9 (BMP9) in osteogenic differentiation of mesenchymal stem cells (MSCs) through RNA interference” [***Genes & Diseases*** 5(2018):172–184]

**DOI:** 10.1016/j.gendis.2023.03.001

**Published:** 2023-03-23

**Authors:** Shujuan Yan, Ruyi Zhang, Ke Wu, Jing Cui, Shifeng Huang, Xiaojuan Ji, Liping An, Chengfu Yuan, Cheng Gong, Linghuan Zhang, Wei Liu, Yixiao Feng, Bo Zhang, Zhengyu Dai, Yi Shen, Xi Wang, Wenping Luo, Bo Liu, Rex C. Haydon, Michael J. Lee, Russell R. Reid, Jennifer Moriatis Wolf, Qiong Shi, Hue H. Luu, Tong-Chuan He, Yaguang Weng

**Affiliations:** a Ministry of Education Key Laboratory of Diagnostic Medicine and School of Laboratory Medicine, Chongqing Medical University, Chongqing 400016, China; b Molecular Oncology Laboratory, Department of Orthopaedic Surgery and Rehabilitation Medicine, The University of Chicago Medical Center, Chicago, IL 60637, USA; c The School of Pharmacy and the Affiliated Hospitals of Chongqing Medical University, Chongqing 400016, China; d Key Laboratory of Orthopaedic Surgery of Gansu Province and the Department of Orthopaedic Surgery, The Second Hospital of Lanzhou University, Lanzhou, Gansu 730030, China; e Department of Biochemistry and Molecular Biology, China Three Gorges University School of Medicine, Yichang, Hubei 443002, China; f Department of Surgery, The Affiliated Zhongnan Hospital of Wuhan University, Wuhan, Hubei 430071, China; g Department of Orthopaedic Surgery, Chongqing Hospital of Traditional Chinese Medicine, Chongqing 400021, China; h Department of Orthopaedic Surgery, Xiangya Second Hospital of Central South University, Changsha, Hunan 410011, China; i Department of Surgery, Laboratory of Craniofacial Biology and Development, Section of Plastic Surgery, The University of Chicago Medical Center, Chicago, IL 60637, USA

The authors regret having several image assembly errors. Specifically, in Figure 3A panel *b*, the image for “AdsimB9-4 only” group was erroneously duplicated with an overlapping image from the “AdRFP” group; and the image for “AdsimB9-1+BMP9” group was erroneously duplicated with an overlapping image from “AdsimB9-8+BMP9” group. In Figure 4A panel a, the images for “BMP9” group and “BMP9+simB9-4” group were erroneously duplicated with an overlapping image from “simB9-4” group. In Figure 5A, the image for “BMP9+simB9-4/Day3” group was erroneously duplicated with an overlapping image from “BMP9+simB9-7/Day3” group; and the image for “BMP9+simB9-4/Day5” group was erroneously duplicated with an overlapping image from an unrelated experiment. In Figure 6B, the image for “BMP9+simB9-7/Day 11” group was erroneously duplicated with an overlapping image from the “BMP9+simB9-4/Day 11” group.

We confirm the errors are restricted to the image assembly, and the underlying data and conclusions are correct and unchanged.

The authors would like to apologize for any inconvenience caused.

**Figure 3** Adenovirus-mediated expression of simB9 siRNAs significantly diminishes exogenous BMP9-induced ALP activity in MSCs. **(A)** Qualitative ALP assay. Subconfluent iMADs were infected with the indicated adenoviruses. At day 5 **(a)** and day 7 **(b)**, the infected cells were subjected to histochemical staining of ALP activity. Each assay condition was done in duplicate. Representative results are shown. **(B)** Quantitative ALP assay. Subconfluent iMADs were infected with the indicated adenoviruses. At days 3, 5, and 7, the infected cells were subjected to bioluminescence assay of ALP activity. Each assay was done in triplicate. “∗” *P* < 0.05, “∗∗” *P* < 0.01 compared with the AdBMP9-infected iMADs group.

**Figure:**
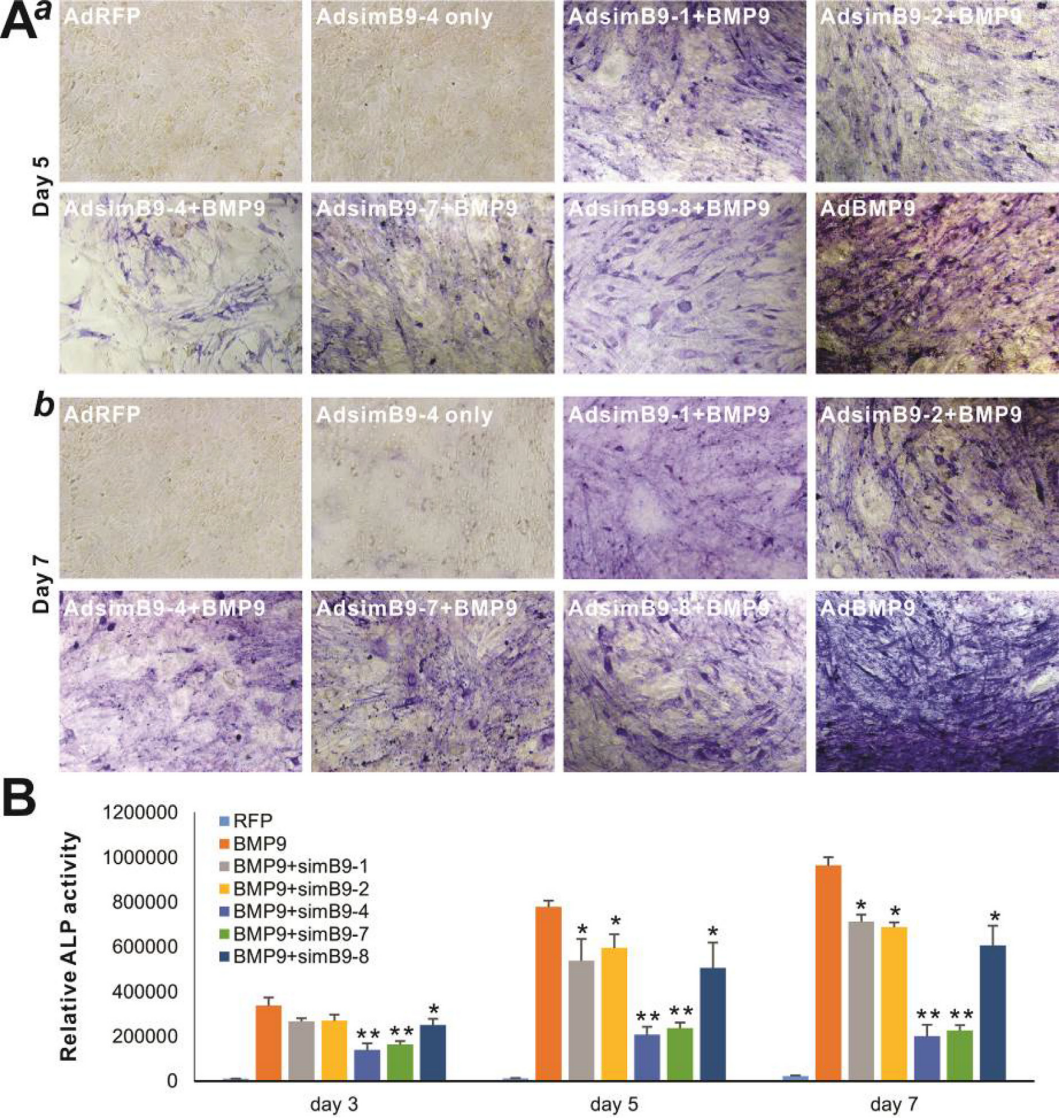

**Figure 4** BMP9-induced expression of osteogenic regulators and biomarkers is effectively inhibited by the co-expression of simB9-4 and simB9-7 siRNAs in MSCs. **(A)** Efficient knockdown of exogenous BMP9 by co-expression of simB9-4 and simB9-7. Subconfluent iMADs were infected with the indicated adenoviruses for 48 h **(a)** and total RNA was isolated from the cells and subjected to qPCR analysis of mouse Bmp9 expression **(b)**. All samples were normalized with Gapdh. Each assay condition was done in triplicate. “∗” *P* < 0.05, “∗∗” *P* < 0.01 compared with the AdBMP9-infected group. **(B)** BMP9-induced expression of osteogenic regulators and biomarkers is effectively inhibited by simB9-4 and simB9-7 siRNAs. Subconfluent iMADs were infected with the indicated adenoviruses. Total RNA was isolated from the cells at day 3 (for Runx2 and Osx) and day 5 (for Opn, Ocn and Bsp), and subjected to qPCR analysis of mouse gene expression. All samples were normalized with Gapdh. Each assay condition was done in triplicate. “∗” *P* < 0.05, “∗∗” *P* < 0.01 compared with the AdBMP9-infected group.

**Figure:**
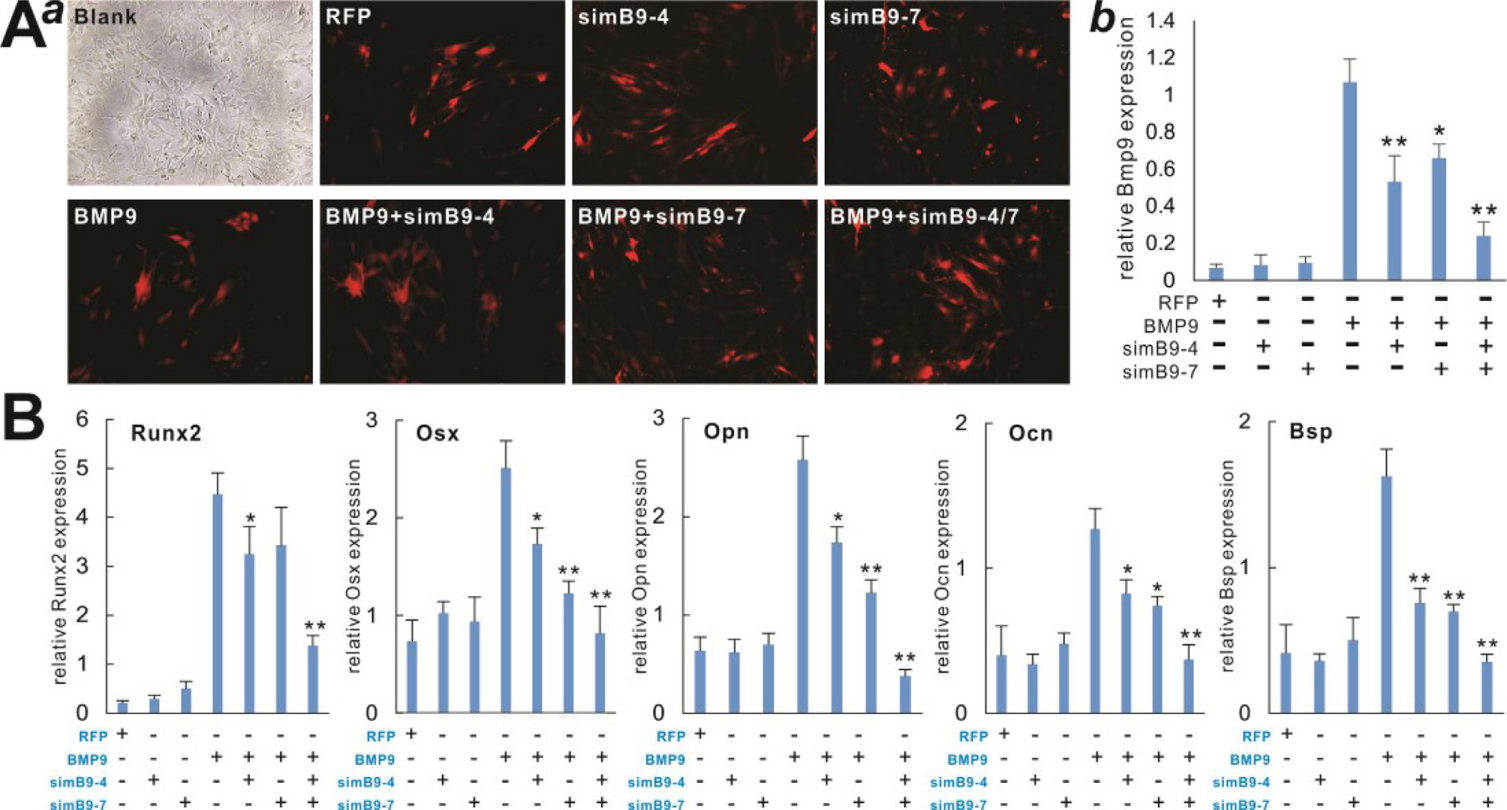

**Figure 5** BMP9-induced ALP activity is effectively inhibited by the co-expression of simB9-4 and simB9-7 siRNAs in MSCs. Subconfluent iMADs were infected with the indicated adenoviruses. At days 3, 5, and 7, the infected cells were subjected to ALP activity assays by either histochemical staining **(A)**, or quantitative bioluminescence assay **(B)**. Each assay was done in triplicate. Representative staining results are shown. “∗” *P* < 0.05, “∗∗” *P* < 0.01 compared with the AdBMP9-infected iMADs group.

**Figure:**
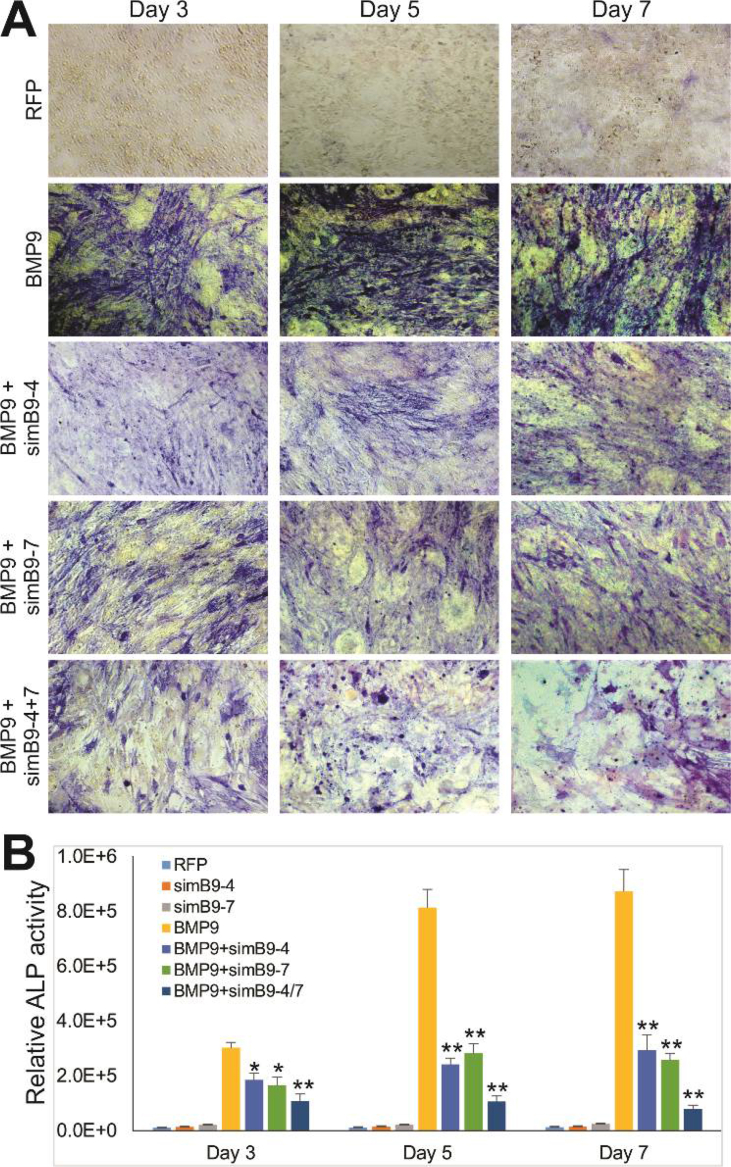

**Figure 6** BMP9-induced matrix mineralization is effectively inhibited by the co-expression of simB9-4 and simB9-7 siRNAs in MSCs. Subconfluent iMADs were infected with the indicated adenoviruses. At days 7 and 11, the infected cells were subjected to Alizarin Red S staining. Staining results were recorded macrographically **(A)** or under a low power microscope **(B)**. Each assay condition was done in duplicate. Representative staining results are shown.

**Figure:**